# Innovating using mHealth for people with dementia and co‐morbidity and their carers: Screening and economic assessment at the population level

**DOI:** 10.1002/alz.088343

**Published:** 2025-01-09

**Authors:** Cecilia Anza‐Ramirez, Maria Kathia Cardenas, Filipa Landeiro, Rosa Montesinos, Nilton Custodio, Marco Da Re, Rafael A Calvo, Veronica Belon‐Hercilla, María Sofía Cuba, Antonio Bernabe, Christopher Butler, Jaime Miranda, IMPACT Dementia Study Group

**Affiliations:** ^1^ CRONICAS, Universidad Peruana Cayetano Heredia, Lima Peru; ^2^ University of Oxford, Oxford United Kingdom; ^3^ Research unit, Instituto Peruano de Neurociencias, Lima Peru; ^4^ Imperial College London, London United Kingdom; ^5^ Universidad Peruana Cayetano Heredia, Lima, Lima Peru; ^6^ The George Institute for Global Health, Sydney Australia

## Abstract

**Background:**

Dementia is a global health challenge, especially in low‐ and middle‐income countries like Peru, where diagnosis, access, and awareness are limited. Within the IMPACT Dementia project, a component focuses on developing, testing, and implementing an mHealth‐enabled system for dementia screening and diagnosis, and assessing its cost‐effectiveness.

**Methods:**

**Phase I. To develop a dementia diagnostic screening mHealth system**: The features of the screening tool will be locally adapted using a co‐design approach including community members and community health workers (CHW) in four regions in Peru (Lima, Tumbes, Iquitos, and Huancayo) to enhance adaptability and efficacy. The final tool will be applied to older adults (60+) in the community by CHW.

**Phase II. To assess the accuracy and acceptability, and characterization of older adults (60+) at the population level in Peru (n≈32,000)**: Using the mHealth system developed in Phase I, assessments including AD8, RUDAS‐PE, and PFAQ, will be conducted. Accuracy for dementia diagnosis will be determined through gold‐standard assessments involving interviews, neuropsychological testing, and the CDR in a subgroup of participants conducted by neurologists. The system will also collect data on socio‐demographic information, carers, co‐morbidities, quality of life, resource usage and costs.

**Phase III. To integrate the mHealth diagnostic system into Peru’s primary healthcare (PHC) level**: After validation, a 3‐month quasi‐experimental study in 2 centres per region will assess system performance, using waiting room recruitment and provider‐conducted screenings for older adults. Indicators will evaluate integration success, sensitivity, and specificity of the tool, cost‐effectiveness, and stakeholder perspectives through a process evaluation, guiding future scalability.

**Result:**

Early phases suggest that the IMPACT Dementia project will offer insights into dementia challenges in Peru. The co‐design phase prioritises cultural relevance. Phase II anticipates a comprehensive dataset characterising older adults, informing prevalence and risk factors, and validating the mHealth diagnostic system. Phase III aims to assess the integration feasibility of the diagnostic system into PHC.

**Conclusion:**

This component of IMPACT Dementia addresses the challenges of dementia in Peru, emphasizing diagnosis, comorbidity, and resource optimization through mHealth. The participation of CHW means a strategic change, which aims to alleviate the burden on health systems.

